# Sustainable Multi-Cycle Physical Recycling of Expanded Polystyrene Waste for Direct Ink Write 3D Printing and Casting: Analysis of Mechanical Properties

**DOI:** 10.3390/polym16243609

**Published:** 2024-12-23

**Authors:** Rubén García-Sobrino, Alejandro Cortés, José Ignacio Sevilla-García, Marta Muñoz

**Affiliations:** Department of Applied Mathematics, Materials Science and Engineering and Electronic Technology, Universidad Rey Juan Carlos, Calle Tulipán s/n, 28933 Móstoles, Spain; ruben.sobrino@urjc.es (R.G.-S.); ji.sevilla.2020@alumnos.urjc.es (J.I.S.-G.)

**Keywords:** circular economy, plastic waste recycling, expanded polystyrene, additive manufacturing, Direct Ink Write

## Abstract

This work investigates the sustainable reuse of expanded polystyrene (EPS) waste through a multi-cycle physical recycling process involving dissolution in acetone and subsequent manufacturing via Direct Ink Write (DIW) 3D printing and casting. Morphology and mechanical properties were evaluated as a function of the manufacturing technique and number of dissolution cycles. Morphological analysis revealed that casted specimens better replicated the target geometry, while voids in 3D-printed specimens aligned with the printing direction due to rapid solvent evaporation. These voids contributed to slightly reduced stiffness in 3D-printed specimens compared to casted ones, particularly for transverse printing orientation. The defoaming process during dissolution significantly increased the density of the material, as well as removed low molecular weight additives like plasticizers, leading to a notable enhancement in stiffness. Successive dissolution cycles led to increased removal of plasticizers, enhancing stiffness up to 52 times (cast), 42 times (longitudinally printed), and 35 times (transversely printed) relative to as-received EPS waste. The glass transition temperature remained unchanged, confirming the preservation of polymer integrity. This work highlights the potential of EPS inks for sustainable, multi-cycle recycling, combining enhanced mechanical performance with the flexibility of 3D printing for complex, cost-effective designs, aligning with circular economy principles.

## 1. Introduction

The growing environmental impact of expanded polystyrene (EPS) waste has driven the need for innovative recycling methods. The current state of research in EPS recycling highlights several methodologies based on thermal, chemical, and physical methods [1]. Thermal recycling of EPS offers a way to convert waste into energy but requires careful management to be sustainable at both environmental and economic levels in terms of its energy requirements [2,3,4]. On the other hand, chemical recycling produces new materials that match the quality of conventional virgin materials [5] and may include processes that convert plastic waste into, for instance, carbon nanomaterials [6]. Finally, physical recycling involves physically processing waste EPS into new products without altering its chemical structure.

The latter methodology, i.e., physical recycling, has been prioritized according to the European Commission to minimize environmental impact and promote sustainable waste management practices across its territory [7]. In contrast to chemical recycling, physical recycling has gained attention for its ability to treat more complex plastic waste streams than the former, but it remains an energy-intensive process and is currently not economically viable on a large scale. In this sense, the European strategy aims to make all plastic packaging on the EU market reusable or recyclable by 2030 [8]. This plan aims to envisage a drastic reduction in the use of single-use plastics, including polystyrene products such as the one cited in this manuscript [9]. Thus, the reduction in plastic packaging is targeted at a percentage decrease of at least 50% by 2025 and 55% by 2030 [9]. These regulations encourage the development and adoption of physical recycling technologies to meet the EU’s circular economic goals; however, this technique may be limited by the degradation of material properties over multiple recycling cycles [10].

In this sense, Ferrándiz-Mas et al. [11] evaluated the reuse of EPS to produce lightweight cement mortars containing up to 60% recycled EPS with mechanical properties comparable to cement, making them suitable for specific applications. Some studies explore the dissolution of EPS in acetone, focusing on the kinetics and composition of the resulting swollen polystyrene used as a binder for molding compounds [12]. There are also studies based on the use of more environmentally sustainable solvents such as limonene [13] or natural essences [14].

On the other hand, 3D printing, or additive manufacturing (AM), is emerging as a revolutionary technology in the field of plastics recycling [15,16,17]. The customization and flexibility that it offers compared to traditional manufacturing technologies, as well as the innovation involved, could facilitate social awareness that promotes the development of a circular economy. To the best of our knowledge, there are several studies in the literature on the recycling of EPS with AM, all of them based on the Fused Filament Fabrication (FFF) technology [18,19,20,21]. Briefly, the operation of this technology consists of the extrusion of a thermoplastic type of filament with the addition of temperature. In this way, the extruded material exits towards a bed or deposit through a nozzle, building the final material layer by layer [22]. However, as mentioned above, the temperature required for the aforementioned extrusion process can lead to high energy consumption on an industrial scale. The requirement of a temperature addition conditions the development of a sustainable recycling process.

Unlike the aforementioned works, this manuscript is based on Direct Ink Writing (DIW). This technology is also an extrusion-based 3D printing technology; however, unlike the filament required by the described FFF technology, DIW starts from a viscous paste-based material that is extruded after pressure from a die. To this end, the above-mentioned viscosity allows the printing of elements at or just above room temperature and atmospheric pressure [23,24]. This quality, ideal for our goal, also favors the printing of other materials such as polymers, waxes, hydrogels, ceramics, and even increasing metals [25], which significantly increases the likelihood of recycling, as confirmed by A. Mantelli et al. [26]. In this sense, the work of M. Zheng et al. [27] presents a new type of photothermal polyurethane (PU) that can be recycled and repaired in situ.

In the case of this work, our group was a pioneer in using 3D printing inks based on physically recycled EPS waste for DIW technology [28]. This work presented a novel and accessible method to any user that allows the physical recycling of EPS using acetone as a solvent without damaging the macromolecules [29]. In that work, the use of this EPS-ink for DIW 3D printing was demonstrated and optimized by printing linear circuits suggesting the possibility of an additive manufacturing system minimizing energy and resource consumption, offering sustainable and cost-efficient methods for producing high-value, complex objects. Nevertheless, just one recycling cycle was studied.

Once the efficacy of this technology was validated through simple geometries and a single recycling cycle, the present work aims to extend this analysis by evaluating the feasibility of using this technology for a greater number of recycling cycles, as well as their effect on the mechanical properties. Controversial hypotheses exist regarding the long-term mechanical integrity and environmental impact of recycled EPS products. Some studies indicate polymer chain degradation by a reduction in both molar weight and glass transition temperature, suggesting an oxidative degradation process [30], while others claim that using as dissolution agents for EPS demonstrates no adverse effects on polymer chain integrity [31]. For this purpose, the effect of conducting three subsequent dissolution and manufacturing cycles with the EPS waste on the mechanical and physicochemical properties was evaluated. Furthermore, the effect of the manufacturing process on the mechanical properties was also studied. Here, the performance of the as-received EPS waste is compared to the one reached by cast and 3D-printed specimens using the physically recycled EPS inks.

## 2. Materials and Methods

### 2.1. Materials

In the present work, the EPS waste was obtained from different centers associated with the Rey Juan Carlos University (Madrid, Spain). It is worth mentioning that the EPS waste was washed and dried when it was collected to avoid any contamination. It should also be noted that this material (washed and dried EPS waste) was labeled as “as received” throughout the manuscript. On the other hand, acetone solvent (99.6%) from GLR Labkem was used for defoaming and minimizing the EPS waste, obtaining the EPS inks for the subsequent manufacturing processes described below.

### 2.2. Preparation of the EPS Inks

**Figure 1:**
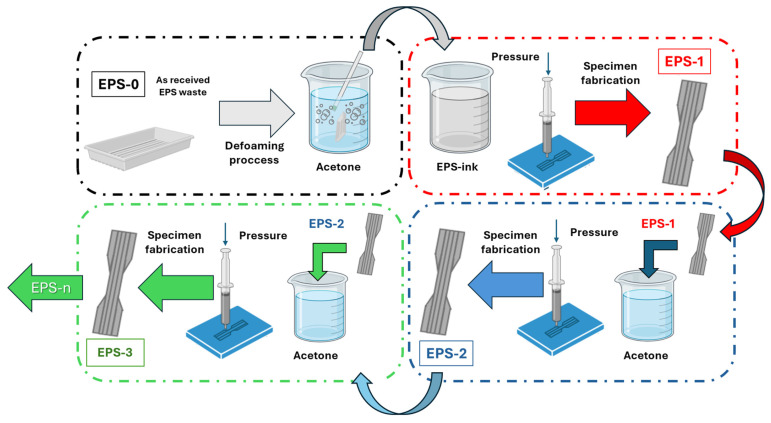
Scheme of the proposed multi-cycle physical recycling process. The as-received EPS waste (EPS-0) was dissolved in acetone, inducing the defoaming process, thus obtaining an EPS-ink (EPS-1). After manufacturing with this ink, the specimens were dissolved again in acetone, obtaining EPS-2, and so on, completing “n” cycles.

The process for obtaining the EPS ink consists of a defoaming process by dissolving the EPS waste in acetone solvent in a 100:1 volume ratio. This process involves the formation of viscous paste suitable for 3D printing using DIW technology. To demonstrate the circularity of the process, three subsequent dissolution and manufacturing cycles were carried out on EPS waste in this research work. Therefore, at this point, it is necessary to define the names of the different EPS products. Here, the as-received EPS waste (EPS-0) is dissolved in acetone to produce EPS-1. Then, after manufacturing and testing specimens with this ink, they were dissolved again in acetone, obtaining EPS-2. Repeating the cycle once more, defoaming and manufacturing yielded EPS-3. Figure 1 illustrates the manufacturing and dissolution processes used to produce the different EPS inks.

### 2.3. Manufacture of Testing Specimens

In this research work, the effect of the number of dissolution cycles on the mechanical properties was studied as a function of the manufacturing process. Two different manufacturing processes were compared (casting and 3D printing) for the EPS-1, EPS-2, and EPS-3 inks. In addition, the mechanical properties were compared to the as-received EPS waste (EPS-0).

**Figure 2:**
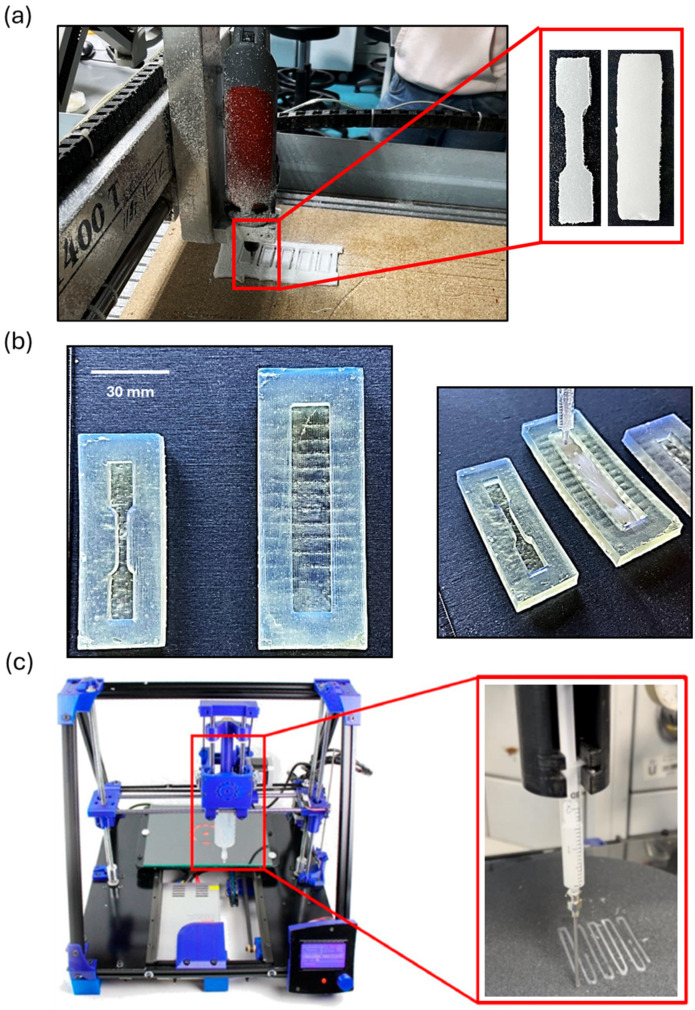
(**a**) CNC machining of the as-received EPS waste (EPS-0) (**b**) Molds obtained by SLA 3D printing technology and casting process using EPS-1, EPS-2, and EPS-3. (**c**) DIW 3D printing technology using a BCN3D Plus printer with a modified Paste Extruder Module.

The specimens of the as-received EPS waste (EPS-0) were obtained by CNC machining using a HIGH-Z S-1400 T CNC-Technik equipment associated with the Mach3 Loader software (see Figure 2a). The machining conditions were as follows: spindle speed of 2000 rpm, 2000 feed rate, and 3 mm of pass depth (stepover 0.5 mm), using a milling cutter with a diameter of 4 mm.

On the other hand, molds with the desired geometries were designed and printed using stereolithography (SLA) 3D printing technology (Formlabs 3B) in Elastic 50A V1 resin using 100 µm of layer height. After the printing process was completed, the molds were washed with 2-isopropanol (99.9%) supplied by Merck and then post-cured by UV radiation using Form Cure equipment for 40 min at 60 °C. Once the molds were obtained, a casting process was conducted (see Figure 2b) using the EPS-1, EPS-2, and EPS-3 inks.

For the 3D-printed specimens, a BCN3D Plus (Barcelona, Spain) DIW 3D printer, with a modified Paste Extruder module, was used in the present study. The latter element was modified to allow printing from disposable syringes, facilitating the accessibility of the technology presented (see Figure 2c). This added element was also printed with High-Impact Polystyrene (HIPS) filament from SmartFil by Fused Filament Fabrication (FFF) 3D printing technology using a BQ Witbox printer. It is worth mentioning that the specimens were printed on SiC-P120 sandpaper (QATM Quality Assured; R_a_ = 24.05 ± 1.27 µm) to improve the adhesion with the printed material and minimize the nozzle drag. Most of the 3D printing parameters for the DIW technology, set up using the Slic3r software, were optimized according to a previously published work [28]. More specifically, a 2.11 mm diameter needle (BENECRAFT), a printing speed of 12 mm/s, an extrusion multiplier of 2, a retraction of 0.5 mm, and a printing bed temperature of 25 °C. Furthermore, the nozzle tip was dipped in silicone oil before printing, acting as a lubricant to reduce friction forces. In this research work, the infill percentage was optimized to match the dimensions of the 3D model in two printing orientations, longitudinal and transverse, regarding the longest dimension of specimens. For this task, 15 × 35 mm^2^ rectangular pieces were printed using infill percentages ranging from 10 to 100%, and the morphology of the resulting specimens was evaluated.

All the 3D models for 3D printing were designed using CATIA v5 software (Dassault Systèmes, Velizy-Villacoublay, France).

### 2.4. Physico-Chemical Characterization

Viscosity characterization was carried out at 25 °C according to ISO-3219 rotational viscosity [32] with a Brookfield DV2T viscometer from AMETEK Brookfield Inc (MA, USA). The spindle used was N-LV3 (63) [33] in the range from 0.1 to 0.4 revolutions per minute (r.p.m.). The viscosity analyses were performed in triplicate.

Weight-average and number-average molecular weights (Mw and Mn, respectively) and the polydispersity index (PDI) were measured by Gel Permeation Chromatography (GPC) with the use of three columns of PL Gel Olexix. The system was calibrated with a narrow molecular weight distribution of standard polystyrene.

Differential Scanning Calorimetry (DSC) tests were carried out using a DSC25 device from TA Instruments (New Castle, DE, USA) from 30 to 200 °C at a 20 °C/min heating rate in a nitrogen atmosphere. The glass transition temperature (Tg) was calculated using the turning point analysis method by TA Instruments TRIOS v5.1.1.46572 software according to ISO-11357 [34].

The density of the samples has been measured by using an analytical balance (Mettler Toledo) with the XPR/SSR-Ana density kit in distilled water at room temperature (20 °C) following the UNE-EN ISO 1183-1 standard [35]. The Archimedes density method was employed to assess the density of the recycled EPS under study. The results were compared with the density obtained for the as-received EPS waste. Each specimen was weighed in air and then submerged in distilled water to calculate the Archimedes density (*D*), using the Equation (1):

(1) $$D=\frac{A}{A-B}\left(D_{0}-D_{l}\right)+D_{l}$$

where (*A*) represents the weight of the sample measured in air; (*D*_0_) is the density of the distilled water in which the part is submerged, taking its temperature into account; (*Dl*) is the air density under room conditions with a value of 0.0012 g/mL; and (*B*) is the weight of the sample when immersed in water.

### 2.5. Mechanical Properties

The mechanical properties were analyzed in tensile and 3-point bending load conditions. In both cases, the universal testing machine used was a Zwick Z100 equipment from ZwickRoell, using a load cell of 5 kN. The dimensions and calculations were conducted according to ASTM D638 [36] for the tensile tests, which were carried out at 5 mm/s speed, whereas the 3-point bending tests were evaluated according to ASTM D790 [37] with a test speed of 10 mm/s. Finally, it should be noted that all studies were conducted in quintuplicate.

## 3. Results and Discussion

### 3.1. Optimization of the 3D Printing Process

**Figure 3:**
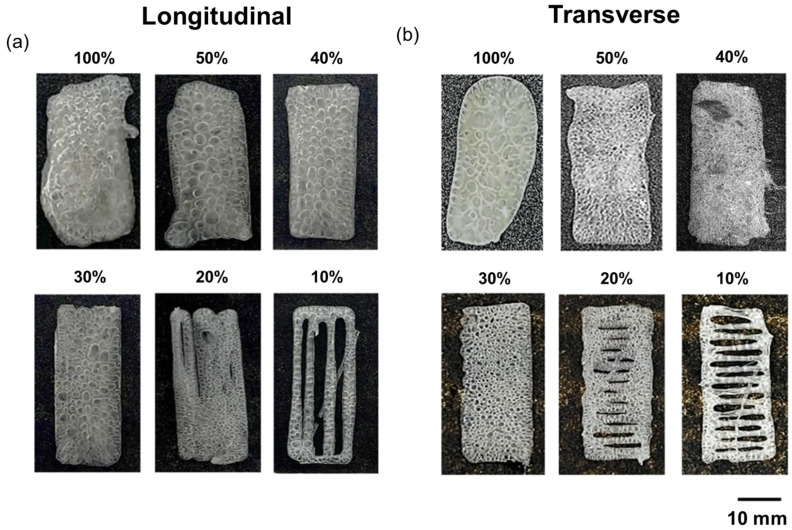
Representative images of the specimens used for the infill percentage optimization study, manufactured in the longitudinal (**a**) and transverse (**b**) printing orientations.

Optimization of the infill percentage of the 3D printing process was conducted to match the dimensions of the 3D model in the two proposed printing orientations (longitudinal and transverse). Figure 3a,b show representative images of the specimens used for the infill percentage optimization study, manufactured in the longitudinal and transverse printing orientations, respectively.

In both cases, a high infill percentage results in geometric distortions relative to the 3D model due to an excess of extruded volume, as clearly observed in specimens with 50% and 100% infill percentages. Conversely, a low infill percentage leads to insufficient filling, resulting in discontinuous specimens, as seen in those with 10% and 20% infill.

Table 1 shows the quantitative dimensional study by measuring and comparing the dimensions of the 3D-printed specimens to the 3D model carried out by image analysis using the continuous specimens (infill percentage above 20%). As previously mentioned, the increase in the infill percentage led to a higher width and length caused by the excess of extruded material. Furthermore, the excess of extruded material yielded a higher geometrical distortion, which was calculated as the standard deviation in length and width. At this point, it is important to highlight the significant presence of voids, regardless of the printing orientation or infill percentage. As demonstrated in our previous research work, these voids are the result of the evaporation of acetone, whose vapor is trapped in the printed sample due to the formation of a thin, dry film on its surface [28]. The kinetics of this process depend on the volume of material expelled [38].

In summary, considering that a higher infill percentage leads to an excess of extruded material and an increased acetone vapor void formation, which promotes geometric distortions in the specimens compared to the 3D model, the lowest infill percentage that produced a continuous specimen, 30%, was selected as the optimum.

### 3.2. Morphological Analysis

Once the repeatability of the 3D printing technique for obtaining solid two-dimensional systems was optimized, the mechanical properties were evaluated as a function of the manufacturing technique and the number of dissolution cycles in acetone. In addition, the influence of the layer orientation of the 3D-printed specimens on the mechanical properties was also studied.

**Figure 4:**
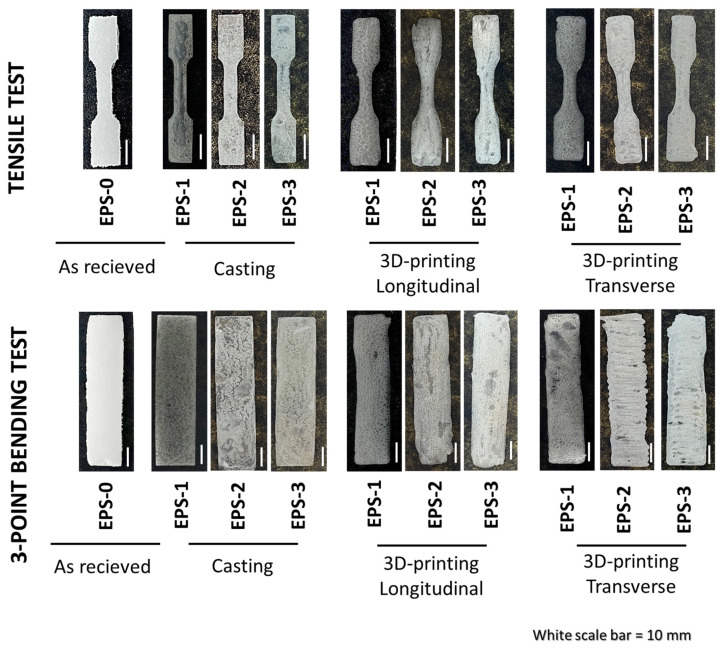
Parts obtained for the tensile and the 3-point bending tests with the different manufacturing methods: CNC machining of as-received EPS waste (EPS-0), casting (EPS-1 to 3), and 3D printing (EPS-1 to 3) in the two proposed printing orientations, longitudinal and transverse.

First, Figure 4 shows the parts obtained with the different manufacturing processes and the number of dissolution cycles. Here, it is worth mentioning the noticeable change in the look and physical properties of the manufactured parts when carrying out the defoaming process (EPS-1) from the as-received EPS waste (EPS-0). Furthermore, the repeatability of the manufacturing processes when conducting the subsequent dissolution cycles is noteworthy, showing the specimens similar dimensions and looks, regardless of the number of dissolution cycles. This illustrates the sustainability of the proposed material since it is possible to recycle the parts several times when reaching their end-of-life, as it is a physical recycling process [31].

When analyzed in detail, the casted specimens replicate the desired geometry better than the 3D-printed ones, which can be ascribed to the inherent nature of DIW 3D printing [39]. Unlike casting, there are no molds in DIW, resulting in slight distortions in the specimen walls. In addition, the syringe drags part of the extruded material nearby when printing, leading to shape distortions, especially when sudden changes in the printing direction occur.

Finally, the void distribution is different between the manufacturing conditions. More specifically, the voids are randomly distributed in the casted specimens, whereas the 3D-printed ones present aligned porosity in the direction of the layer orientation. This can be explained due to the rapid evaporation of the solvent. In contrast to casting, where the viscous paste fills the mold uniformly, the volume of the printed part is filled by extruding continuous parallel lines in DIW 3D printing. The solvent evaporates almost instantly from the printed ribbon surface during printing, generating a thin, solid skin that impedes ink flow with the subsequent parallel ribbon. Therefore, both the possible entrapped air between ribbons and the voids grown inside each printed ribbon would remain aligned in the printing direction. The effect of the aforementioned features on the mechanical properties will be further discussed.

### 3.3. Mechanical Properties as a Function of the Manufacturing Technology

**Figure 5:**
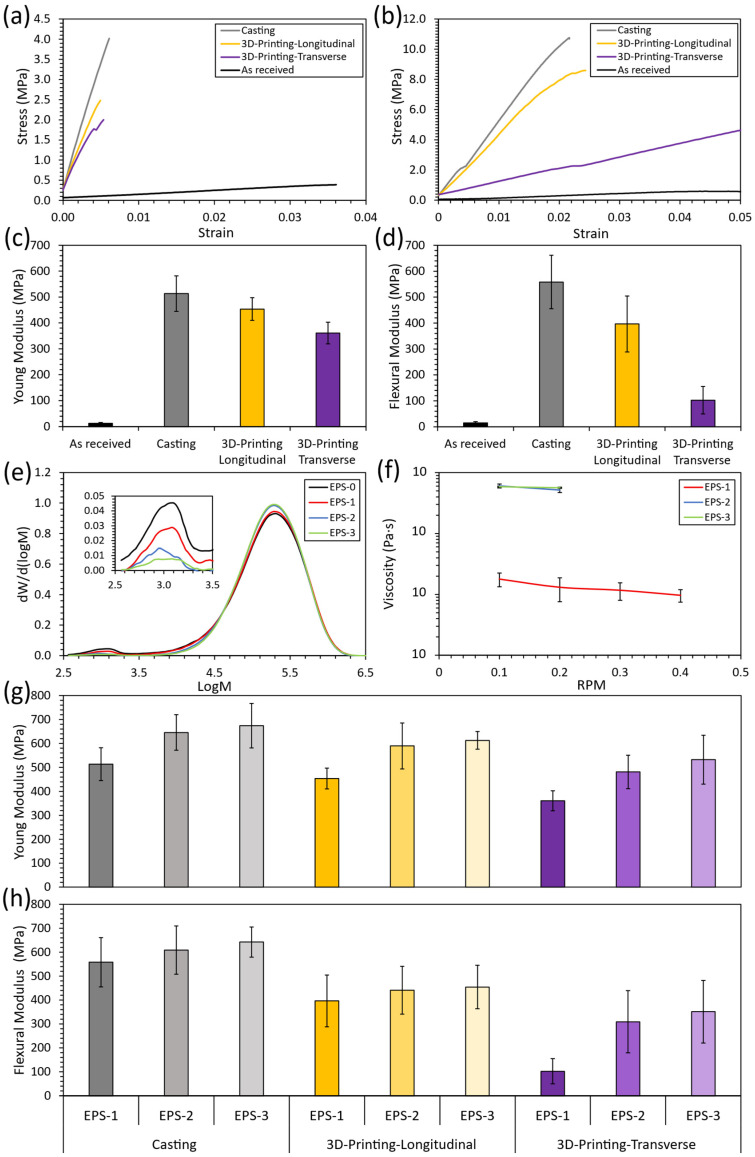
Example curves of tensile (**a**) and three-point bending tests (**b**). Young’s (**c**) and flexural (**d**) modulus as a function of manufacturing technology, EPS-0 for the as-received material and EPS-1 for the casted and 3D-printed specimens. Molecular weight distribution obtained by GPC (**e**) and viscosity (**f**) as a function of the number of dissolution cycles in acetone. Young’s (**g**) and flexural (**h**) modulus as a function of manufacturing technology and the number of dissolution cycles in acetone.

Figure 5a,b show representative stress–strain curves obtained from the tensile and 3-point bending tests, respectively, as a function of the manufacturing process, where the as-received specimens were obtained using the EPS-0, and the casting and 3D-printed specimens were obtained using EPS-1, after conducting the defoaming process by dissolution in acetone solvent. Here, all specimens obtained with EPS-1 exhibited significantly increased mechanical properties compared to the received EPS waste, demonstrating higher stiffness and tensile strength, despite suffering a marked decrease in ductility, particularly under tensile loading.

More specifically, Figure 5c,d show an in-depth analysis of stiffness, which is the most useful property when comparing brittle materials [40] for the tensile and 3-point bending tests, respectively [41,42].

As previously mentioned, a significant increase in stiffness is shown by the casted and 3D-printed specimens. These results can be ascribed to the defoaming process of the as-received EPS waste, which increases density and removes some additives, such as plasticizers. The increase in density when dissolving the as-received EPS waste in acetone is shown in Table 2. Here, the density increases around one order of magnitude, confirming that the defoaming process has taken place. In addition, no considerable differences were observed when increasing the number of dissolution cycles, evincing that the defoaming process is completed from the first dissolution cycle. When comparing the density of the physically recycled specimens with the density of bulk polystyrene (0.96–1.05 g/mL), a lower density is obtained, which can be attributed to the void growth caused by solvent evaporation. The removal of additives, such as plasticizers, was demonstrated in previous research work [28] and evinced again in this work by GPC analysis (see Figure 5e). Here, the decrease in the peak ranging from 2.5 to 3.5 logM indicates the removal of a component with low molecular weight, which was associated with the removal of additives. This point will be further discussed.

However, analyzing the mechanical properties in detail, the decrease in the mechanical properties of the 3D-printed specimens is also noticeable concerning the casting ones, which can be explained by the internal structure of the specimens. As previously mentioned in Section 3.2, the ink flow between adjacent ribbons in the 3D-printed specimens is hindered due to the rapid formation of a thin, dry film on each ribbon’s surface when extruded, leading to a lack of cohesion and entrapped air between adjacent ribbons. Finally, when comparing the two different printing orientations, the specimens printed in the transverse orientation exhibited a lower stiffness than the ones printed in the longitudinal orientation. These results support the assumptions about the rapid formation of the thin, dry film on the ribbon’s surface when extruded since the specimens printed in the transverse orientation present more parallel ribbons, leading to the previously mentioned lack of cohesion and entrapped air between adjacent ribbons. Thus, the manufacturing defects are, in this case, aligned perpendicular to the applied forces both in the tensile and three-point bending tests, resulting in a decrease in the mechanical properties [43,44].

It is worth noting that, despite the reduction in the mechanical properties of the 3D-printed specimens compared to the casted ones, stiffness increased on average by more than 36 times for those printed in the longitudinal orientation and over 28 times for those printed in the transverse orientation, relative to the as-received EPS waste (average values obtained using the EPS-1 ink under tensile and three-point bending load conditions). Furthermore, this increase in stiffness, combined with the advantages of 3D printing, enables the fabrication of more complex or customized parts, including unique designs, without significantly increasing production costs. Unlike traditional manufacturing methods, such as casting, 3D printing does not require molds that need to be amortized, making it a more flexible and cost-effective solution for producing intricate geometries or tailored components.

### 3.4. Mechanical Properties as a Function of the Number of Dissolution Cycles

To study the mechanical properties as a function of the number of dissolution cycles, it is essential to first examine how these cycles affect the properties of the EPS ink. Figure 5e shows the GPC analysis as a function of the dissolution cycles in acetone, whereas Table 3 summarizes the main results obtained from GPC and DSC analyses. Here, the decrease in the peak ranging from 2.5 to 3.5 logM when increasing the number of dissolution cycles is again related to the remotion of polymer additives, such as plasticizers, presenting low molecular weight. This is also directly related to the decrease in the polydispersity index (PDI) and the increase in Mw and Mn, pointing out that the polymer chain length distribution becomes more homogeneous when increasing the number of dissolution cycles. Furthermore, the DSC analysis shows that the glass transition temperature is not affected by the number of dissolution cycles, according to our previous study, since this is a physical recycling process that does not cause damage to the polymer chains as no chemical reactions are involved [28,29].

However, the removal of polymer additives when increasing the number of dissolution cycles significantly increases ink viscosity, as shown in Figure 5f, which could hinder the manufacturing process. Despite this, both the removal of additives and the increase in viscosity tend to stabilize from the second dissolution cycle (EPS-2) onward. Furthermore, the increase in the number of dissolution cycles enhances stiffness in both tensile and three-point bending load conditions, as shown in Figure 5g,h, respectively, again related to the remotion of additives, such as plasticizers. More specifically, the stiffness increased on average by more than 52 times for the cast specimens, over 42 times for those 3D-printed in the longitudinal orientation, and over 35 times for those printed in the transverse orientation, in comparison to the as-received EPS waste (average values obtained using the EPS-3 ink under tensile and three-point bending load conditions).

In this sense, considering that this maximum of viscosity, reached in the third dissolution cycle (EPS-3), does not impede manufacturing by casting and is within the printability limits of DIW 3D printing, no limitations in the number of dissolution cycles are expected, evincing the sustainability of the proposed inks based on EPS waste.

Furthermore, it is worth highlighting the projection of the proposed technology in terms of circular economy and sustainability. In addition to significantly reducing the volume of EPS waste with a minimum amount of solvent (100:1 volume ratio) while enhancing mechanical properties, the process is fully scalable. In fact, any company or home user that has access to a conventional FFF 3D printing machine can freely download files to cheaply manufacture and assemble a DIW device to conduct the proposed recycling process, thus giving a new useful life to EPS waste and obtaining on-demand custom or even unique parts considering the advantages of additive manufacturing. Likewise, acetone is a common solvent, affordable, and easy to acquire, which further facilitates its scalability.

## 4. Conclusions

The present research work focuses on optimizing the reuse of expanded polystyrene waste (EPS-0) through a physical recycling process involving dissolution in acetone and subsequent manufacturing by two methods: DIW 3D printing and casting. This study explores the influence of 3D printing parameters, such as infill percentage and printing orientation, on the morphological and mechanical properties of the fabricated specimens. Additionally, the impact of multiple recycling cycles on ink properties, including viscosity and molecular structure, as well as the mechanical performance of the resulting components, was evaluated. The ink obtained in the first dissolution cycle was named EPS-1, whereas those obtained in the second and third dissolution cycles were named EPS-2 and EPS-3, respectively.

Regarding the optimization of 3D printing parameters, the optimal infill percentage for 3D printing was determined to be 30%, as it provided continuous specimens without geometric distortions or insufficient filling. A higher infill percentage (≥50%) caused geometric distortions due to excessive extruded material, while a lower infill percentage (≤20%) resulted in discontinuities. Then, the morphological analysis showed that the casted specimens replicated the desired geometry more accurately than the optimized 3D-printed ones, likely due to the absence of molds in 3D printing and material dragging during deposition. Voids were randomly distributed in cast specimens but aligned with the printing direction in 3D-printed specimens, stemming from the rapid formation of a thin, dry film on the extruded ribbon’s surface during printing, hindering ink flow between adjacent printed ribbons.

This fact explains the slight decrease in mechanical properties of the 3D-printed specimens compared to the casted ones, especially for the ones printed in the transverse orientation due to the alignment of defects perpendicular to the applied forces in the latter. The increase in the number of dissolution cycles resulted in a higher removal of low molecular weight additives like plasticizers, leading to increased stiffness. The glass transition temperature remained unaffected (around 104 °C), confirming that the physical recycling process preserved polymer integrity. Stiffness improved with successive dissolution cycles, achieving a maximum enhancement of 52 times (casted), 42 times (longitudinally printed), and 35 times (transversely printed) compared to as-received EPS waste (12.5 ± 3.2 MPa in tensile load conditions) due to the increase in density (in a factor of 10) related to untreated EPS waste (0.05 ± 0.03 g/mL), caused by the defoaming process.

Despite the decrease in stiffness of 3D-printed parts compared to those casted, 3D printing offers advantages over casting by enabling the fabrication of complex or customized parts without molds, reducing production costs, especially for intricate designs.

Viscosity increased with additional cycles, stabilizing from the second cycle (EPS-2) onward without impeding manufacturability. Therefore, the physical recycling process demonstrates a sustainable approach to reusing EPS waste, allowing for multiple life cycles of the material while significantly enhancing mechanical properties.

## Figures and Tables

**Table 1:** Quantitative dimensional study.

**3D Model**					
Width (mm)		15			
Length (mm)		35			
**3D-printed—Longitudinal**					
	Infill (%)	100	50	40	30
Width	Average width (mm)	16.32 ± 1.66	16.15 ± 1.39	15.55 ± 0.57	15.61 ± 0.32
	Width deviation (%)	10.17	8.65	3.66	2.04
Length	Average length (mm)	35.97 ± 2.16	35.46 ± 0.96	35.83 ± 0.41	35.76 ± 0.28
	Length deviation (%)	6.01	2.71	1.14	0.78
**3D-printed—Transverse**					
	Infill (%)	100	50	40	30
Width	Average width (mm)	14.87 ± 1.88	15.92 ± 0.67	15.64 ± 0.90	15.84 ± 0.27
	Width deviation (%)	12.64	6.17	5.75	1.70
Length	Average length (mm)	36.02 ± 3.66	35.77 ± 1.05	35.86 ± 1.15	35.61 ± 0.44
	Length deviation (%)	10.16	2.93	3.2	1.23

**Table 2:** Density study as a function of the number of dissolution cycles in acetone.

Sample	Density (g/mL)
EPS-0	0.05 ± 0.03
EPS-1	0.52 ± 0.03
EPS-2	0.51 ± 0.03
EPS-3	0.52 ± 0.02

**Table 3:** GPC and DSC analysis as a function of the number of dissolution cycles in acetone.

Value/Label	EPS-0	EPS-1	EPS-2	EPS-3
Mw (g/mol) × 10^4^	23.85	24.46	24.26	24.75
Mn (g/mol) × 10^4^	2.77	4.25	6.42	7.66
PDI	8.61	5.75	3.78	3.23
Tg (°C)	103.08	102.95	104.39	104.76

## Data Availability

Data are available upon request.
